# The Role of Genomic Data in the Discovery, Annotation and Evolutionary Interpretation of the Interferon-Lambda Family

**DOI:** 10.1371/journal.pone.0004933

**Published:** 2009-03-20

**Authors:** Brian A. Fox, Paul O. Sheppard, Patrick J. O'Hara

**Affiliations:** Bioinformatics Department, ZymoGenetics, Inc, Seattle, Washington, United States of America; BMSI-A*STAR, Singapore

## Abstract

**Background:**

Type-I interferons, type-II interferons, and the IL-10 family are helical cytokines with similar three-dimensional folds. However, their homologous relationship is difficult to detect on the basis of sequence alone. We have previously described the discovery of the human type-III interferons (IFN lambda-1, -2, -3 or IL-29, IL-28A, IL-28B), which required a combination of manual and computational techniques applied to predicted protein sequences.

**Principal Findings:**

Here we describe how the use of gene structure analysis and comparative genomics enabled a more extensive understanding of these genes early in the discovery process. More recently, additional mammalian genome sequences have shown that there are between one and potentially nine copies of interferon lambda genes in each genome, and that several species have single exon versions of the interferon lambda gene.

**Significance:**

The variable number of single exon type-I interferons in mammals, along with recently identified genes in zebrafish homologous to interferons allows a story of interferon evolution to be proposed. This model suggests that the gene duplications and single exon retrotransposons of mammalian type-III interferons are positively selected for within a genome. These characteristics are also shared with the fish interferons and could be responsible for the generation of the IL10 family and also the single exon type-I interferons.

## Introduction

The use of sequence similarity between proteins to infer a functional relationship helped to define the beginnings of bioinformatics. Algorithms that rapidly compare large volumes of protein or DNA sequences have often been used to explore evolutionary relationships between genes and between organisms (e.g. BLAST [1]). Concurrently, the richness and complexity of the genome has motivated study of the non-coding regions, including: chromosome rearrangement, transcription factor binding sites, repetitive and transposable elements, exon-intron gene structure, comparative genomics and genetic variation, among others. The study of non-coding regions has allowed the development of additional approaches to identify evolutionary relationships between genes.

Many groups have pursued the identification and functional annotation of novel mammalian helical cytokines (interleukins and interferons) based on protein sequence similarity. However, helical cytokines are notoriously hard to detect based on protein sequence alone because their three-dimensional fold is particularly permissive to sequence perturbation [2]. This makes distantly homologous sequences difficult to distinguish from non-homologous sequences of similar amino acid content. A quasi-threading approach was developed to specifically recognize the protein sequence for members of this family [3], [4]. Later developments to this technique include using a combination of protein similarity and exon-intron gene structure to characterize many diverse members of the helical cytokine family [5], and to identify evolutionary subclasses within this family. However, these techniques still generate many false positives which lead to expensive efforts to try to validate the computational discovery in the wet lab. Therefore, we have used a combination of protein sequence and genomics based methods to focus our lab efforts on good candidate cytokines and drop wasted efforts on the false positives.

Several years ago, we and our colleagues discovered a novel helical cytokine that we first described in a patent application entitled “Interferon Like Protein” [6]. Later, we described two other proteins that were members of the same ligand family (initially referred to as the IL28 family), whose members were designated IL29 (the protein in ref. [6]), IL28A, and IL28B by HUGO and a heterodimeric class II cytokine receptor for all three proteins, IL10-RB/IL28Rα [7]. Simultaneously, Kotenko et. al. [8] described the same receptor (which they designated IFN-λR1), and demonstrated that the protein described in ref. [6] was a ligand for this receptor. They also described the other two members of the ligand family, and designated the three ligands interferon λ1 (the protein in ref. [6]), λ2 and λ3 (HUGO aliases: IFNL1, IFNL2, IFNL3). Both of these papers [7], [8] also described the signaling activity of the ligands and the expression pattern of the receptor.

These genes have recently been classified as type-III interferons by the Nomenclature Committee of the International Society for Interferon and Cytokine Research, and the lambda nomenclature will be used here. Protein sequence similarity shows that the interferon lambda family is approximately equidistant between type-I interferons and the IL10 cytokine family. One receptor subunit is shared between the heterodimeric receptors for interferon lambda and IL10, and there is commonality in the gene structures of the interferon lambda and IL10 families. Because the type-III interferons show anti-viral activity [6], [7], [8], [9], [10], [11], [12], and the distribution of their receptor complex is distinct from the type-I IFN receptor [7], [8], [13], interferon lambda has the potential for *in vivo* anti-viral activity with fewer side-effects than interferon alpha. ZymoGenetics is currently conducting clinical trials of PEGylated IFNL1 for the treatment of chronic Hepatitis C infection.

Previously [7], we provided a brief description of how we discovered this gene family. Specifically, we compiled a set of appropriately sized (140–200 residues), potentially secreted proteins [14] from human gene predictions [15], and with no readily detectable sequence similarity to other known genes. Families of similar proteins within this smaller set were found, and the helical cytokine quasi-threading method [4] and manual protein alignments allowed this novel cytokine family to be identified. In this paper, we will describe the genomic data that provided further evidence for interferon-lambda membership in the helical cytokine family. The more recent availability of many mammalian genomes has allowed us to identify members of the interferon-lambda family; the diversity of these family members has some interesting parallels with the type-I interferon family. The parent gene of the interferon lambda gene family may be the same parent gene which gave rise to the type-I interferons and the IL10 family. The discovery and annotation of the interferon-lambda family is a case where many pieces of bioinformatics analysis built upon each other to discover a very exciting new sub-family within the drug and target rich family of helical cytokines.

## Analysis

### Protein Sequence

The similarity between any of the human interferon lambda proteins to any other helical cytokine or interferon is below the detection limit for BLAST [16], even using a very permissive p-value threshold. A manual sequence alignment reveals that the specific percent identity between the interferon lambda family and other helical cytokine families is approximately 5% (to type-II interferon), 12% (to type-I interferons), and 15% (to IL10 family members). This weak similarity and the corresponding lack of BLAST detection is consistent with the relationships between the type-I interferons, type-II interferon and the IL10 families, i.e. the inter-family percent identity between any of these families is between 5 and 12%. Within the human interferon lambda family, the percent identity is between 81% and 96%, with IFNL2 and IFNL3 being the most closely related.

Given the inability to detect any similarity using BLAST, other methods were required for discovery. Using the quasi-threading technique [4], proteins can be given a score based on how compatible their sequence is with the helical cytokine fold. At the time of its discovery [6], [7], the human interferon-lambda family members scored at a level such that approximately 400 proteins from human REFSEQ score as well as this family, yet only about 13% of these higher scoring proteins are known helical cytokines. Furthermore, 10% of the helical cytokines known at the time of discovery had a lower score than the interferon lambda family. As described in Sheppard et. al. [7], a multiple sequence alignment of interferon lambdas with IL10 and type-I interferons increased our confidence in the quasi-threading score because it showed that the amphipathic helices, flexible loops and some cysteine locations appeared to be consistent between type-III interferons and the other helical cytokines.

### Exon-intron structure

**Figure 1:**
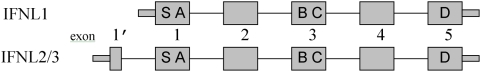
Gene structure of human IFNL1, IFNL2 and IFNL3. Each numbered box represents an exon (not drawn to scale). The shorter extensions at the terminal exons represent noncoding DNA. The letters inside the boxes are protein structural features: (S)ecretion peptide, helix (A), helix (B), helix (C) and helix (D).

The type-III interferon family has a multi-exon gene structure (Figure 1) in which there are five conserved exons and an initial exon which is observed in IFNL2 and IFNL3 but not IFNL1. However, the original gene predictions for human IFNL2 and IFNL3, produced by GENSCAN [15], contained only five exons. The cloned cDNA sequence of human IFNL2 and IFNL3 mRNA revealed an unpredicted upstream exon (exon 1'), as shown in Figure 1 and reported earlier [7]. This exon contains a short coding region of only four amino acids, which includes the codon for the initiating Met. As opposed to the other introns in this gene which are all phase zero, the intron after exon 1' splits a codon such that one nucleotide of the codon triplet resides on exon 1'. All other exon boundaries between IFNL2 and IFNL3 are in the same positions as those in IFNL1. Interestingly, a multi-exon interferon from zebrafish has a differentially expressed transcript with an alternate splice event at the 5′ end in which an additional exon eliminates the secretion signal [17]. In summary, the human genes in the type-III interferon family have five conserved exons, but there is an additional 5′ exon in IFNL2 and IFNL3 which codes for a few amino acids, including the initial Met.

### Gene family evolution: genomic organization and species conservation

In the completed mammalian genomes of human and mouse, the type-III interferon genes are organized in a single gene cluster and in a very similar pattern [18]. The gene ordering in human (chr 19q13) and mouse (chr 7A3) is nearly identical and has been described [18], where the nomenclature has been established as follows: interferon-lamba-1, interferon-lamba-2, interferon-lamba-3 and interferon-lamba-4-pseudo, with the first pair on the reverse strand, and the second pair on the forward strand. However, interferon-lambda-1 is a pseudogene in mouse, while interferon-lambda-4 is a pseudogene in both species [18] (experimental cloning of human IFNL4 was attempted in our company and several spliced transcripts were found in various tissues [data not shown]; however, 5′ RACE experiments did not reveal an ORF with a Met, which led us to conclude that human IFNL4 is a pseudogene). In both human and mouse, approximately 6 kilobases (kb) of DNA at gene loci for IFNL2 and IFNL3 have nucleotide sequence identity greater than 97% for the whole genomic region (including exons, introns and ∼1 kb flanking DNA). In both genomes, these two gene loci are inverted with respect to each other and have about 10 kb of intervening DNA [18]. When human IFNL2 and IFNL3 are compared to IFNL1, only the coding and regulatory regions share any significant DNA similarity.

We used the human members of the interferon lambda family to query most of the completed and draft genomes in the ENSEMBL database (version 49, March 2008) using BLAST. Full length or unique fragments of type-III interferon family members were found in 23 of 24 of the mammalian species and in chicken, but not in the 7 other eukaryotic species that we checked (Table 1). Specifically, we used tblastn (protein query and DNA database) with the default search parameters (word size = 3, BLOSUM62 matrix, gap insertion/extension = 9/2, using the ENSEMBL BLAST interface, these parameters are closest to the “distant homologies” or “no optimisation” search) and found the mammalian hits to have significance scores (P-values) better than about 10E-9. For the multiple exon gene loci, we browsed the hits and examined the ends of the putative exons and also manually verified that the different hits within a single genome were distinct sequences. There were a varying number of gene copies, with the same exon pattern and/or full length single exon genes for each species with type-III interferon genes. In all cases where interferon lambda genes have multiple exons, the predicted exon boundaries are conserved. One organism, the common shrew, has up to 9 distinct members of this family. However, since many of these genomes are of draft quality, some of the gene hits which appear to be different gene loci may actually be the same gene locus but either have errors or are only partially sequenced. For example, in the common shrew, 5 of the 9 possible gene loci were found on contigs which are long enough to contain the full length sequence. The 5 loci were very similar to each other, with about 97% identity in the coding regions and 87% identity in the introns. For the remaining 4 gene loci in common shrew, the contigs were shorter and only contained one end of the coding region, which implies that those 4 regions may be as few as 2 different genes with the middle part of the gene not yet sequenced and assembled. Furthermore, depending on the finished sequence, the gene loci counts listed in Table 1 may include pseudogenes, since some of the BLAST results showed that a frameshift would be necessary to remain in the correct reading frame. To computationally predict if any of these genes are expressed, we searched dbEST [19] using BLAST and only found a handful of human ESTs and a single EST from pig (EW341616). Overall, the mammals contain a wide range in the number of copies of interferon lambda gene loci.

**Table 1:** Number and gene structure of Interferon Lambda genes in many genomes from ensembl v49.

Species (brief name)	Maximum number of multi-exon gene loci [a] (and chromosomal location, if known)	Number of single exon gene loci (and chromosomal location, if known)	Number of nearly full length genes (and pseudogene, if confirmed)	genome sequence version (in ENSEMBL 49)
Armadillo	-	-	-	ARMA
Bat	5*	2	1	MICROBAT1
Bush baby	1	-	-	BUSHBABY1
Cat	4*	1	3	CAT
Chimp	4 (c19)	-	1	CHIMP2.1
Cow	-	1* (c13)	1	Btau_3.1
Dog	2 (c1)	1 (c24)	3	BROADD2
Elephant	2	-	-	BROADE1
Guinea pig	3	-	1	GUINEAPIG
Hedgehog	5	-	-	HEDGEHOG
Horse	3* (c22)	1 (c10)	3	EquCab2
Human	4 (19q13)	-	3 (1)	NCBI36
Lemur	3	-	-	micMur1
Mouse	3 (7A3)	-	2 (1)	NCBIM37
Opossum	2 (c4, c3)	-	-	BROADO5
Orangutan	6 (c19)	-	1	PPYG2
Pika	5*	-	-	pika
Platypus	3	-	-	OANA5
Rabbit	2	-	-	RABBIT
Rat	4 (c1, c17)	-	1	RGSC3.4
Rhesus	6* (c19)	-	1	MMUL_1
Shrew	9	-	5	COMMON_SHREW1
Squirrel	2*	-	-	SQUIRREL
Tree shrew	2	-	1	TREESHREW
Chicken	1 (c7)	-	-	WASHUC2
Frog	-	-	-	JGI4.1
Fugu	-	-	-	FUGU4
Zebra fish	-	-	-	ZFISH7
C elegans	-	-	-	WS180
Fly	-	-	-	BDGP5.4
Mosquito	-	-	-	AgamP3
Yeast	-	-	-	SGD1.1

[a] To be consistent, this total includes genes and pseudogenes. For draft genomes, not finding all exons in a short contig due to assembly and sequencing gaps is indistinguishable from a pseudogene with some exons missing.

* the noted species has one gene which needs a frameshift of 1 bp or 2 bp needed in order to stay in the “correct” reading frame. This frameshift might indicate that this is a pseudogene, or it might be a sequencing error due to the draft nature of the genome sequence.

There were also many examples of type-III interferon genes which are contained in a single coding exon. For example, in dog, there were three type-III interferon family genes in which each gene locus was fully contained on its own contig of draft genomic sequence; but, none of the genes were on the same contig. One of the dog genes was located on chromosome 24 and has a single predicted exon (also see [20]), and the other two are multi-exon genes which are neighbors of unknown distance and orientation on chromosome 1. In dog, the single exon gene is 80% identical to one of the multi-exon genes; whereas, the two multi-exon genes are 70% identical to each other. In all species where the chromosomal location is available for both types of genes, the single exon genes are located on a different chromosome than the multi-exon genes (Table 1), suggesting a retrotransposition event for the gene duplication [21], [22].

The most distant organism in which our BLAST queries of human type-III interferon against the ENSEMBL genomes were able to detect orthologs was chicken. Despite the fact that this search did not find any hits in fish, frog or invertebrates, genes coding for interferon family members have been identified in fish [23], [24], [25], [26], [27]. These genes contain five exons and had been originally grouped with the type-III interferons based on protein similarity [23]. However, other analyses [28], [20], and our own sequence comparisons conclude that the amino acid sequences of interferons from fish are considerably more similar to mammalian type-I interferons. Thus, it appears that fish interferons have the same multi-exon gene structure as mammalian type-III interferons, yet their protein sequence is more similar to type-I interferons.

**Figure 2:**
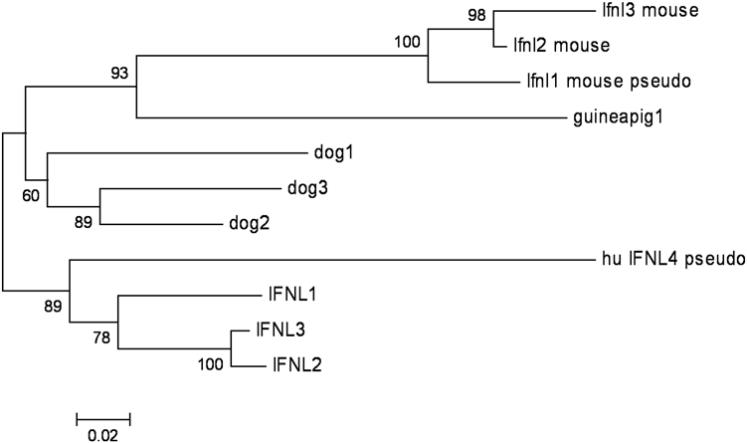
Phylogenetic tree using the actual or predicted DNA sequences of spliced interferon lambda genes or pseudogenes in human, mouse, dog and guinea pig. Human and mouse gene loci are labeled as in Lasfar et. al. [18]. The labels for dog and guinea pig are not intended to be suggestions for permanent names. The “dog1” gene is the single exon gene from dog on chromosome 24, while “dog2” and “dog3” are multi exon genes located near each other on chromosome 1 (see Supplementary Figure S1 for gene sequences). The sequence for the more distant mouse pseudogene, named interferon lambda 4 in Lasfar et. al., was excluded because we were unable to find the locus. The optimal tree is drawn to scale, with bootstrap values shown at branch points and branch lengths in the same units as those of the evolutionary distances used to infer the phylogenetic tree (sum of branch length = 1.0194). All positions containing gaps and missing data were eliminated from the dataset, and the full codons were used. There were a total of 314 nucleotide positions in the final dataset, compared to the longest sequence of 622 nucleotides (the pseudogenes shortened the total number of columns which were available for alignment).

We generated a phylogenetic tree with the human, mouse, dog and guinea pig genes, including the human and mouse pseudogenes (Figure 2, and Supplementary Figure S1). The reason we used a nucleotide alignment which included the pseudogenes was so that we could determine the relative ages of the pseudogenes. Due to the degeneracy of the genetic code, and the observation that nucleotide substitutions occur at differing rates for each type of nucleotide, there are more detailed theoretical models of estimating evolutionary distances with nucleotide sequences than with protein sequences. The tree was made using the neighbor-joining method [29] in MEGA4 [30] with evolutionary distances estimated by Tamura-Nei [31]. In an effort to better estimate the robustness of the trees which were generated, bootstrapping [32] was also performed (N = 1000), in which variants of the input alignment are used to create trees which are compared to the initial tree. The Tamura-Nei distance was chosen for Figure 2 because it is a popular method; however, the tree structure and bootstrap values were very robust and did not change appreciably as different distance measures were used, or as the codon positions used in the distance were varied. In the resulting tree for interferon lambda in human, mouse, dog and guinea pig, the bootstrap values indicate that the mouse and human genes and pseudogenes are reliably separate from each other.

## Discussion

Due to the sequence diversity of the helical cytokine fold, the type-III interferons are very difficult to detect as homologs using standard bioinformatics tools. As reported earlier [7], only the combined use of specialized software to detect a helical cytokine fold [4], other protein sequence filters (length, secretion signal, sequence clustering into a novel family), and manual knowledge-based multiple sequence alignments, allowed us to identify these family members as novel helical cytokines. However, even these methods generate false positives, and, we used additional methods based on genomic information to add confidence to our assignment of sequences to the helical cytokine family. The use of genomic, non-coding sequence information to further annotate a gene is an important method in gene discovery, especially when protein similarity methods begin to reach their limit.

The chromosomal locations, exon-intron gene structure and promoter analysis can be used to validate evolutionary relationships between helical cytokines and infer function. For example, in the IL10 family of cytokines, four genes (IL10, IL19, IL20 and IL24) are located in a cluster at human 1q32, and the other two IL10 family members (IL22 and IL26) are located at 12q15 (with IFNG nearby) [5]. The human type-III interferon gene family is also located in its own chromosomal cluster (19q13). Furthermore, all six of the IL10 family genes, along with IL3, IL21 and IFNL1 (IL29) have the identical five exon gene structure in which intron locations divide the alpha helices in analogous positions and no codons are split by introns [5]. Lastly, the promoter of IFNL2 and IFNL3 contains potential immune-related transcription factor binding sites [33]; however, these regulatory regions are not conserved in ancient fish [34]. These genomic insights, along with the species conservation, helped increase our confidence in the importance of the type-III interferon family as functionally important helical cytokines.

In this paper, we show that the 5-exon INFL1 and 6-exon INFL2/3 are very similar to the 5-exon IL10 gene family. As we previously reported [7], INFL2 and IFNL3 have a starting exon with a very short coding region (exon 1'), while the remainder of the introns have the same intron phase and position as IFNL1 and all the members of the IL10 family. In addition, there is evidence in the mouse genome for a potential exon with a splice donor about 100 bp upstream which contains an ATG and two other amino acids in the correct frame (data not shown). Sequences of human IFNL2 and IFNL3 cDNAs were published [8] which do not contain exon 1'; and, similarly, the published sequences of mouse type-III interferons [18] also lack this intron. However, both of those studies used PCR to clone interferon lambda family members from genomic DNA starting at the predicted secretion cleavage site, followed by cloning the spliced mRNA from mammalian cells. Thus, for human IFNL2 and IFNL3, there is no evidence for a transcript which omits exon 1'. Other mammalian mRNA needs to be characterized in order to determine the extent of the presence of exon 1'. It is unclear whether the five- or six- exon version of interferon lambda was present in the rodent-primate ancestor.

Given the desire to make animal model data relevant to humans, we made an effort to determine a probable orthology assignment between the interferon lambda genes in human and mouse. One common bioinformatics approach to determine orthology involves comparing the detailed ordering of related genes on a chromosomal locus between species. Since the interferon lambda gene organization between human and mouse appears so similar, this is the approach that was used to assign the names of the mouse genes in Lasfar et. al. [18]. This approach to orthology assignment assumes that the ancestral species of mouse and human also had a similar gene organization which was conserved for nearly 100 million years and is still present today. However, a phylogenetic tree using the DNA sequences for the human, mouse, dog and guinea pig genes (Figure 2) shows that the primate-rodent speciation event occurred before the multiple gene loci duplicated and diverged from each other. Given this tree (Figure 2), the most parsimonious interpretation is that all current interferon lambda genes arose from a single interferon lambda gene in an ancestral species. Another possibility is that the human-mouse ancestor had four genes arranged like it is observed in humans and that a recent gene conversion event (in both human and mouse) caused all of the genes to appear more closely related. Regardless, the observation that human and mouse have a nearly identical gene ordering requires a recent coincidence in either gene duplication or gene conversion, as it is unlikely that the present day genes are the direct product of a gene duplication or conversion event from a multiple gene cluster in the human-mouse ancestor. Due to the widely varying number of type-III interferon genes in mammalian species, and the observation that in several species these genes are most closely related to others in their same genome, the simplest explanation is all these genes are derived from a single gene in the mammalian ancestor.

With respect to biological function, gene expression patterns and the role of genomic duplication, it is unclear how to interpret the significance of the similar human and mouse gene ordering and the variation in gene count between mammals. Multiple copies of genes allow for higher RNA expression under a greater number of conditions and also for divergence of function. Single exon gene variants, usually the product of a retrotransposition event, are smaller than their multi-exon counterparts which may allow for more frequent and higher fidelity duplication. The order and orientation of genes in compact genomes such as viruses and bacteria can also have large effects on their expression patterns; so, perhaps the similar gene ordering in human and mouse represents some sort of optimum for regulating the expression levels [35], [36]. In an analysis of copy number variants (CNV) and gene duplications throughout the human genome, a recent study [37] has proposed that gene duplications such as CNV occur frequently for all functional families of proteins, but that the duplication of genes encoding environmental response proteins are positively selected over time, as opposed to genes encoding proteins involved in basic cellular processes, which are negatively selected for duplication. We explored the ratios between the total number of type-I interferons and type-III interferons in mammals and could not find any convincing relationship between the total numbers of these genes; nor was there sufficiently clear information regarding viral diversity across these organisms to speculate why some mammals have a much larger repertoire of interferons than others. The long-term persistence of duplicate copies of type-I and type-III interferons is consistent with each of these protein families having environmental-response biological roles.

The evolutionary origins of type-I and type-III interferons are intertwined and both families probably originate from a single interferon gene family present in ancient fish, with representation in present-day fish. Only one family of interferons has been found in present-day fish [17], [27], [28], and there are attempts to argue that the these fish interferons should be classified as type-I interferons or type-III interferons. However, since the fish interferons represent the ancestor gene that gave rise to both families, it is not entirely meaningful to attempt to classify the parent gene as being more related to one child than another. Because of this parental relationship, the interferon in fish has been called interferon-phi [38]. There is much evidence of shared ancestry, since the protein sequence of the fish interferons appears more similar to the type-I interferons, while the multi-exon gene structure is identical to the type-III interferons. Another commonality between the fish interferons and the mammalian type-I family is the cysteine pattern and presence of the conserved CAWE motif [27] – which both differ from the mammalian type-III interferons. One complementary method to determine if the fish interferons are more like type-I or type-III interferons is to identify and characterize the receptors. Levraud et. al. [17] found the heterodimer receptor (zCRFB2 and zCRFB5) in zebrafish which responds to zIFN, and they concluded that the receptors were more like the mammalian interferon type-III receptor complex. However, a more recent paper [27] noted that Levraud et. al. only looked at one of the three potential fish interferon genes. Furthermore, a review of class 2 cytokine evolution [20] successfully predicted the heterodimer receptor (CRFB2 and CRFB5) for zIFN, which they classify as a type-I interferon; and, they further predict that a different pair may serve as an alternate interferon receptor system Overall, the current data suggest the fish interferon family is the evolutionary predecessor to both mammalian interferon families (type-I and type-III), and has gene, protein and biological similarities with each family.

A model of interferon evolution can be developed as follows (also see [20], [23]). Four hundred million years ago, fish contained a single family of interferons. This fish interferon family had multiple exons and its biological role was to respond to viral infection. Different fish species had differing numbers of interferon genes, but they were all closely related. We do not know if the different interferons bound to different receptors or if any species had single exon versions of this gene family. As mammals first appeared about three hundred million years ago, the original interferon family had a genetic event where the spliced RNA was reincorporated into the genome as a retrotransposon. This new single exon gene had the same DNA sequence as the parental gene. Over time, this new family (type-I interferon) kept much of the original function, as evidenced by its slow accumulation of differences compared to the fish interferon family. However, as a single exon gene, it was easily duplicated over time and modern day mammals have a wide range of copies of type-I interferons, perhaps to increase overall expression levels throughout many cell types in the body. However, the original multi-exon gene family, which also gave rise to the IL10 family of cytokines, diverged more quickly from the ancestral gene and eventually became known as type-III interferons. The type-III interferons maintained an important biological role in anti-viral response, and copies arose via gene locus duplication, and in some species, via retrotransposons. Independent duplication events occurred even after different mammalian species diverged. Some duplications in both the type-I and type-III interferon gene families have lead to inactive pseudogenes. For example, in humans, IFNL1 diverged from the IFNL2/3 ancestor after the rodent/human divergence, but long before the recent duplication event between IFNL2 and IFNL3, in which the coding and non-coding regions of the gene duplication are still evident. Present day mammals contain a wide ranging number of copies of type-I and type-III interferons, with the type-III interferons present as both multi-exon and single-exon genes.

In this paper, we have shown the detailed genomic analysis that we used for the initial annotation of these proteins. Further, the recent availability of many draft mammalian genomes has allowed us to notice many parallels in gene duplication and intron-less reinsertion between the type-III interferons and the type-I interferons. The interferon gene family in fish has similarities with both of these mammalian families. The evolutionary story of this dynamic gene family and its role in immunity from fish to mammals is becoming increasingly clear.

## Supporting Information

Figure S1 Nucleotide sequence alignment of actual or predicted interferon lambda genes used in the production of Figure 2.
(0.04 MB DOC)

## References

[1] Altschul S, Madden T, Schaffer A, Zhang J, Zhang Z (1997). Gapped BLAST and PSI-BLAST: a new generation of protein database search programs.. Nucleic Acids Res.

[2] Hill E, Morea V, Chothia C (2002). Sequence conservation in families whose members have little or no sequence similarity: the four-helical cytokines and cytochromes.. J Mol Biol.

[3] Blumberg H, Conklin D, Xu W, Grossmann A, Brender T (2001). Interleukin 20: discovery, receptor identification, and role in epidermal function.. Cell.

[4] Conklin D (2004). Recognition of the helical cytokine fold.. Journal of computational biology : a journal of computational molecular cell biology.

[5] Conklin D, Haldeman B, Gao Z (2005). Gene finding for the helical cytokines.. Bioinformatics.

[6] Sheppard PO, Presnell SR, Fox BA, Gilbert T, Haldeman BA, Grant FJ (2002). Interferon-like protein zcyto21.. Patent WO 0202627-A.

[7] Sheppard P, Kindsvogel W, Xu W, Henderson K, Schlutsmeyer S (2003). IL-28, IL-29 and their class II cytokine receptor IL-28R.. Nature immunology.

[8] Kotenko S, Gallagher G, Baurin V, Antes A, Shen M (2003). IFN-lambdas mediate antiviral protection through a distinct class II cytokine receptor complex.. Nature immunology.

[9] Jordan WJ, Eskdale J, Boniotto. M, Rodia M, Kellner D, Gallagher G (2007). Modulation of the human cytokine response by interferon lambda-1 (IFN-1/IL-29).. Genes and Immunity.

[10] Contoli M, Message SD, Laza-Stanca V, Edwards MR, Wark PA (2006). Role of deficient type III interferon-lambda production in asthma exacerbations.. Nature Medicine.

[11] Ank N, West H, Paludan S (2006). IFN-lambda: novel antiviral cytokines.. Journal of interferon & cytokine research : the official journal of the International Society for Interferon and Cytokine Research.

[12] Uze G, Monneron D (2007). IL-28 and IL-29: newcomers to the interferon family.. Biochimie.

[13] Sommereyns C, Paul S, Staeheli P, Michiels T (2008). IFN-Lambda (IFN-λ) Is Expressed in a Tissue-Dependent Fashion and Primarily Acts on Epithelial Cells In Vivo.. PLoS Pathogens.

[14] von Heijne G (1986). A new method for predicting signal sequence cleavage sites.. Nucleic acids research.

[15] Burge C, Karlin S (1997). Prediction of complete gene structures in human genomic DNA.. J Mol Biol.

[16] Gish W (1996–2004). http://blast.wustl.edu.

[17] Levraud JP, Boudinot P, Colin I, Benmansour A, Peyrieras N (2007). Identification of the zebrafish IFN receptor: implications for the origin of the vertebrate IFN system.. Journal of immunology.

[18] Lasfar A, Lewis-Antes A, Smirnov S, Anantha S, Abushahba W (2006). Characterization of the mouse IFN-lambda ligand-receptor system: IFN-lambdas exhibit antitumor activity against B16 melanoma.. Cancer research.

[19] Boguski MS, Lowe TM, Tolstoshev. CM (1993). dbEST–database for “expressed sequence tags”.. Nature Genetics.

[20] Krause CD, Pestka S (2005). Evolution of the Class 2 cytokines and receptors, and discovery of new friends and relatives.. Pharmacology & therapeutics.

[21] Brosius J, Tiedge H (1995). Reverse transcriptase: Mediator of genomic plasticity.. Virus Genes.

[22] Woelk C, Frost S, Richman D, Higley P, Pond S (2007). Evolution of the interferon alpha gene family in eutherian mammals.. Gene.

[23] Lutfalla G, Roest Crollius H, Stange-Thomann N, Jaillon O, Mogensen K, Monneron D (2003). Comparative genomic analysis reveals independent expansion of a lineage-specific gene family in vertebrates: the class II cytokine receptors and their ligands in mammals and fish.. BMC genomics.

[24] Altmann SM, Mellon MT, Distel DL, Kim CH (2003). Molecular and functional analysis of an interferon gene from the zebrafish, Danio rerio.. Journal of Virology.

[25] Long S, Wilson M, Bengten E, Bryan L, Clem LW (2003). Identification of a cDNA encoding channel catfish interferon.. Developmental and Comparative Immunology.

[26] Robertsen B, Bergan V, Rokenes T, Larsen R, Albuquerque A (2003). Atlantic salmon interferon genes: cloning, sequence analysis, expression, and biological activity.. Journal of Interferon Cytokine Research.

[27] Zou J, Tafalla C, Truckle J, Secombes CJ (2007). Identification of a second group of type I IFNs in fish sheds light on IFN evolution in vertebrates.. Journal of immunology.

[28] Robertsen B (2006). The interferon system of teleost fish.. Fish & shellfish immunology.

[29] Saitou N, Nei M (1987). The neighbor-joining method: a new method for reconstructing phylogenetic trees.. Mol Biol Evol.

[30] Tamura K, Dudley J, Nei M, Kumar S (2007). MEGA4: Molecular Evolutionary Genetics Analysis (MEGA) Software Version 4.0.. Mol Biol Evol.

[31] Tamura K, Nei M (1993). Estimation of the number of nucleotide substitutions in the control region of mitochondrial DNA in humans and chimpanzees.. Mol Biol Evol.

[32] Felsenstein J (1985). Confidence Limits on Phylogenies: An Approach Using the Bootstrap.. Evolution.

[33] Krivan W (2005). Fishing for Proteins in the Pacific Northwest.. http://dx.doi.org/10.1007/b105490.

[34] Venkatesh B, Kirkness EF, Loh YH, Halpern AL, Lee (2006). Ancient noncoding elements conserved in the human genome.. Science.

[35] Hanscombe O, Whyatt D, P Fraser P, Yannoutsos N, Greaves D (1991). Importance of globin gene order for correct developmental expression.. Genes & Development.

[36] Vogel JH, von Heydebreck A, Purmann A, Sperling S (2005). Chromosomal clustering of a human transcriptome reveals regulatory background.. BMC Bioinformatics.

[37] Korbel JO, Kim PM, Chen X, Urban AE, Weissman S (2008). The current excitement about copy-number variation: how it relates to gene duplications and protein families.. Current Opinion in Structural Biology.

[38] Stein C, Caccamo M, Laird G, Leptin M (2007). Conservation and divergence of gene families encoding components of innate immune response systems in zebrafish.. Genome Biology.

