# Supplementary material for: The Role of Genomic Data in the Discovery, Annotation and Evolutionary Interpretation of the Interferon-Lambda Family
Source: PLoS One. 2009 Mar 20;4(3):e4933. doi: 10.1371/journal.pone.0004933 (PMC2654155; doi:10.1371/journal.pone.0004933)
Supplement: Figure S1 — Nucleotide sequence alignment of actual or predicted interferon lambda genes used in the production of Figure 2. (0.04 MB DOC) [file pone.0004933.s001.doc]

Figure S1: Nucleotide Sequence alignment used in the production of Figure 2

dog1 ------------------ATGGCTACAGCTCGGGTCCTGGTGCTGGTGACCGTGGTGCTG

dog2 ---------------------------------GTGCTGGTGCTGACAGCTGTGGTGCTG

dog3 ------------------ATGGCCATTGCATGGGTGCTGGTGCTAGTGACTGCAGGCCTG

guineapig1 ------------------ATGCCGCTGCTTCTGCTGCTGCTGCTGCTGGCCACAGTGCAG

hu_IFNL4_pseudo ------------------------------------------------------------

IFNL1 ------------------ATGGCTGCAGCTTGGACCGTGGTGCTGGTGACTTTGGTGCTA

IFNL2 ATGAAACTAGACATGACTGGGGACTGCACGCCAGTGCTGGTGCTGATGGCCGCAGTGCTG

IFNL3 ATGAAACTAGACATGACCGGGGACTGCATGCCAGTGCTGGTGCTGATGGCCGCAGTGCTG

Ifnl1_mouse_pseudo ------------------------------------------------------------

Ifnl2_mouse ------------------ATGCTCCTCCTGCTGTTGCCTCTGCTGCTGGCCGCAGTGCTG

Ifnl3_mouse ------------------ATGCTCCTCCTGCTGTTGCCTCTGCTGCTGGCCGCAGTGCTG

dog1 GGCTTGACCAGAGCCGGCCCTGTCCCTA---CTTCCAAACCCACCACAACCAGGAGGGGC

dog2 ACGGCGACGGGAGCAGTTCCTTTCCCCAAGCCCCTTGGGGTCCTCGCAGATGCACGGGGC

dog3 AGCTTGGCCAGAGCAGGCCCTGTCCCCA---CTTCCAAGCCCACCACGGCCTGGAGGGGC

guineapig1 ACCAGGACAGGGGCAGTTCCTGTCCCCAGACCAGTCAGTGTCTCTCTGGATACCAAGGAA

hu_IFNL4_pseudo ------------------------------------------------------------

IFNL1 GGCTTGGCCGTGGCAGGCCCTGTCCCCA---CTTCCAAGCCCACCACAACTGGGAAGGGC

IFNL2 ACCGTGACTGGAGCAGTTCCTGTCGCCAGGCTCCACGGGGCTCTCCCGGATGCAAGGGGC

IFNL3 ACCGTGACTGGAGCAGTTCCTGTCGCCAGGCTCCGCGGGGCTCTCCCGGATGCAAGGGGC

Ifnl1_mouse_pseudo ------------------------------------------------------------

Ifnl2_mouse ACAAGAACCCAAGCTGACCCTGTCCCCAGGGCCACCAGGCTCCCAGTGGAAGCAAAGGAT

Ifnl3_mouse ACAAGAACCCAAGCTGACCCTGTCCCCAGGGCCACCAGGCTCCCAGTGGAAGCAAAGGAT

dog1 TGCCACATGGACAGGTTCCAGTCTCTGTCACCCAGGGAGCTAGAAGCCTTCAAGAAGGCC

dog2 TGCCACTTGGCCCAGTTCCAGTCTCTGTCCCCACAAGAGCTGCAGGCCTTCAAGAGGGCC

dog3 TGTGACATTGGCAGGTTCAAATCTCTGTCACCAAGGGAGCTGGAGGCTTTCAAGAAGGCC

guineapig1 TGTCATATGGCCAAGTTCAAGTCTCTGTCCCCACAAGAGCTACAGGCCTTCAAGACGGCC

hu_IFNL4_pseudo ------------------------------------------------------------

IFNL1 TGCCACATTGGCAGGTTCAAATCTCTGTCACCACAGGAGCTAGCGAGCTTCAAGAAGGCC

IFNL2 TGCCACATAGCCCAGTTCAAGTCCCTGTCTCCACAGGAGCTGCAGGCCTTTAAGAGGGCC

IFNL3 TGCCACATAGCCCAGTTCAAGTCCCTGTCTCCACAGGAGCTGCAGGCCTTTAAGAGGGCC

Ifnl1_mouse_pseudo ------------------------------------------------------------

Ifnl2_mouse TGCCACATTGCTCAGTTCAAGTCTCTGTCCCCAAAAGAGCTGCAGGCCTTCAAAAAGGCC

Ifnl3_mouse TGCCACATTGCTCAGTTCAAGTCTCTGTCCCCAAAAGAGCTGCAGGCCTTCAAAAAGGCC

dog1 AAGGATGCCTTGGAAGAGTCGCTCTCCTGGAAGAACTGGAGCTGCAGCTCTCGCCTCTTC

dog2 AAGGACACCTTTGAAGAGTCGCTTTCCCAGAAGGCTTGGAGCTGCCGCCCCCGACTCTTC

dog3 AAGGATGCTTTGGAATATTCACT------AAAAAACTGGAGTTGCAACTCCCGCCTCTTC

guineapig1 AAGGATGCCTTTGAAGAGCAGCTCCTGCTGAAGGACTCCAGGTGCAGCTTGCGCCTCTTC

hu_IFNL4_pseudo ------------GAAGTGTCGCTTCTGCTGAAGGACTGCAGGTGCCGCTCCCGCCTCTTC

IFNL1 AGGGACGCCTTGGAAGAGTCACTCAAGCTGAAAAACTGGAGTTGCAGCTCTCCTGTCTTC

IFNL2 AAAGATGCCTTAGAAGAGTCGCTTCTGCTGAAGGACTGCAGGTGCCACTCCCGCCTCTTC

IFNL3 AAAGATGCCTTAGAAGAGTCGCTTCTGCTGAAGGACTGCAAGTGCCGCTCCCGCCTCTTC

Ifnl1_mouse_pseudo ------------------------------------------------------------

Ifnl2_mouse AAGGATGCCATCGAGAAGAGGCTGCTTGAGAAGGACCTGAGGTGCAGTTCCCACCTCTTC

Ifnl3_mouse AAGGGTGCCATCGAGAAGAGGCTGCTTGAGAAGGACATGAGGTGCAGTTCCCACCTCATC

dog1 CCTAGATCCAGGGACCTGAGACTCCTGCAGGCCTGGGAGCGTCCTGTGGCCTTGGAGGCT

dog2 CCCAGGACCTGGGACCTGCAGCAGCTGAAGGTGTGGGAGCGCCCCATGGCCTTGGAGGCT

dog3 CCTAGAAACCGGGACCTGAGACAGCTACAGGTGTGGGAGCGCCCTGTGGCCTTGGAGGCT

guineapig1 CCCAGGACCTGGGACCTGGGGCAGCTGCAGGTGTGGGAGCGCCCCCTGGCCCTGCAGGCT

hu_IFNL4_pseudo CCCAGGACCTGGGACCTGAGGCAGCTGCAGGTGAGGGAGCGCCCTGTGGCTTTGGAGGCT

IFNL1 CCCGGGAATTGGGACCTGAGGCTTCTCCAGGTGAGGGAGCGCCCTGTGGCCTTGGAGGCT

IFNL2 CCCAGGACCTGGGACCTGAGGCAGCTGCAGGTGAGGGAGCGCCCCATGGCTTTGGAGGCT

IFNL3 CCCAGGACCTGGGACCTGAGGCAGCTGCAGGTGAGGGAGCGCCCCGTGGCTTTGGAGGCT

Ifnl1_mouse_pseudo ---------------------------CAGGTCCAGGAGCACCCCAAGGCCTTGCAAGCT

Ifnl2_mouse CCCAGGGCCTGGGACCTGAAGCAGCTGCAGGTCCAAGAGCGCCCCAAGGCCTTGCAGGCT

Ifnl3_mouse TCCAGGGCCTGGGACCTGAAGCAGCTGCAGGTCCAAGAGCGCCCCAAGGCCTTGCAGGCT

dog1 GAGCTAGACTTGACACTGAAGGTCCTGGAGAACAT------GACTGACTCATCGCTGGGG

dog2 GAGCTGGCCTTGACACTGAAGGTCCTGGAGGCCAT------GGCTGACTCGTCCCTGGGG

dog3 GAGCTGGCCTTGACACTGAAGGTCCTGGAGACCAT------GGCTGACAGGTCCTTGGGG

guineapig1 GAGCTGGCCCTGACCCTGAAGGTCCTGGGCACCGT------GACAGACCCGGCCCTGGAG

hu_IFNL4_pseudo GAGCTGGCCCTGACACTGAAGGTCCTGGAGGTCACCGCTGATGCTGATCCGGCCCTGGGG

IFNL1 GAGCTGGCCCTGACGCTGAAGGTCCTGGAGGCCGC------TGCTGGCCCAGCCCTGGAG

IFNL2 GAGCTGGCCCTGACGCTGAAGGTTCTGGAGGCCACCGCTGACACTGACCCAGCCCTGGTG

IFNL3 GAGCTGGCCCTGACGCTGAAGGTTCTGGAGGCCACCGCTGACACTGACCCAGCCCTGGGG

Ifnl1_mouse_pseudo GAGGTGGCCCTGACCCTGAAGGTCTGGGAGAACAT------AACTGACTCAGCCCTGGCC

Ifnl2_mouse GAGGTGGCCCTGACCCTGAAGGTCTGGGAGAACAT------GACTGACTCAGCCCTGGCC

Ifnl3_mouse GAGGTGGCCCTGACCCTGAAGGTCTGGGAGAACAT------AAATGACTCAGCCCTGACC

dog1 GTGACCCTGGACCAGCCCCTCCGCACGCTGCACCACATCCACTCGGAGCTCCAGGCTTGT

dog2 GACATCCTGGACCAGCCCCTTCACACGCTGCGCCACATCCACTCCGAGCTCCAGGCCTGT

dog3 GACATCCTGGACCAGCCCCTTCACACGCTGCGCCACATCCACTCCCAGCTCCAGGCCTGT

guineapig1 GACATCCTGGAGCAGCCCCTCCACATGCTGCGTCACATCCACGCCCAGCTGCAGGCCTGT

hu_IFNL4_pseudo GATGTCCTGGACCAGCTCCTTCACACCCTCCACAACATCCTCTCCCAGCTTGGGGCCAGT

IFNL1 GACGTCCTAGACCAGCCCCTTCACACCCTGCACCACATCCTCTCCCAGCTCCAGGCCTGT

IFNL2 GACGTCTTGGACCAGCCCCTTCACACCCTGCACCATATCCTCTCCCAGTTCCGGGCCTGT

IFNL3 GATGTCTTGGACCAGCCCCTTCACACCCTGCACCATATCCTCTCCCAGCTCCGGGCCTGT

Ifnl1_mouse_pseudo ACCATCCTGGGCCAGCCTCTTCACACACTGAGCCACATCCACTCCCAGCTGCAGACCTGT

Ifnl2_mouse ACCATCCTGGGCCAGCCTCTTCATACACTGAGCCACATTCACTCCCAGCTGCAGACCTGT

Ifnl3_mouse ACCATCCTGGGCCAGCCTCTTCATACACTGAGCCACATTCACTCCCAGCTGCAGACCTGT

dog1 GT---------CCCGGCTCAGCCCACAGCAGACCCCAGGCCCC---ACGGCCGCCTCCAC

dog2 GTGACTCAGCCCCCAGCTCAGCCCCCAGCAGGCTCCCGGCCCC---GAGGCCGCCTCCAC

dog3 G---------------CTCAGCCCCCAGCAGGTCCCCAGCCCC---GAGGCCGCCTCCAC

guineapig1 GT---------GCCCACCCTGCGCACCGCAGCCCCCAGGCCCGTCAGCCGCCGCCTGTCC

hu_IFNL4_pseudo GT---------GAATCC--AGCCT-CAGCAAGGCCCAGGCCCC---GGGGCCGCCTCCAC

IFNL1 AT---------CCAGCCTCAGCCCACAGCAGGGCCCAGGCCCC---GGGGCCGCCTCCAC

IFNL2 AT---------CCAGCCTCAGCCCACGGCAGGGCCCAGGACCC---GGGGCCGCCTCCAC

IFNL3 AT---------CCAGCCTCAGCCCACGGCAGGGCCCAGGACCC---GGGGCCGCCTCCAC

Ifnl1_mouse_pseudo AC---------ACAGCCTCAGCCCACAGCAGAGCACAGGCCCCTGAGCCGCCACCTCTCC

Ifnl2_mouse AC---------ACAGCTTCAGGCCACAGCAGAGCCCAGGTCCCCGAGCCGCCGCCTCTCC

Ifnl3_mouse AC---------ACAGCTTCAGGCCACAGCAGAGCCCAAGCCCCCGAGTCGCCGCCTCTCC

dog1 CACTGGCTGCACCGGCTCCAGAAGGCCCCCAAG---GAGTCCCAGGGCTGCCTCGAGGCC

dog2 CACTGGCTGCAGCGCCTCCATGATGCCCCAGAGAAGGAATCCCTCGGCTGCCTGGAAGCC

dog3 CCCTGGCTGCATCGGCTCCATGAGGCCTCAAAGAAGGAGTCTCAAGGCTGCCTCGAAGCC

guineapig1 CACTGGCTGCAGAGGCTCAACCAGGCCTCCAAGAAGGCGTCCCCTGGCTGCCTCCAGGAG

hu_IFNL4_pseudo CACTGGCTGCACCAGCTCCAGGAGGCCCCGAGGAAGCA-TCACTGGAATTATAGGCATGA

IFNL1 CACTGGCTGCACCGGCTCCAGGAGGCCCCCAAAAAGGAGTCCGCTGGCTGCCTGGAGGCA

IFNL2 CATTGGCTGTACCGGCTCCAGGAGGCCCCAAAAAAGGAGTCCCCTGGCTGCCTCGAGGCC

IFNL3 CATTGGCTGCACCGGCTCCAGGAGGCCCCAAAAAAGGAGTCCCCTGGCTGCCTCGAGGCC

Ifnl1_mouse_pseudo CGCTGGCTGCACAGACTCCAGGAGGCCCAGAGTAAGGAGACCCCTGGCTGCCTGGAGGCC

Ifnl2_mouse CGCTGGCTGCACAGGCTCCAGGAGGCCCAGAGCAAGGAGACCCCTGGCTGCCTGGAGGCC

Ifnl3_mouse CGCTGGCTGCACAGGCTCCAGGAGGCCCAGAGCAAGGAGACTCCTGGCTGCCTGGAGGAC

dog1 TCCATCACGTTCAACCTCTTCCGCCTCCTCACACGGGACCTGAAATGTGTTGCCAGTAGA

dog2 TCTGTCATGTTCAACGTCTTCCGCCTCCTCACCCGGGACCTGAAATGTGTGGCCAGTGGA

dog3 TCTGTCCTCTTCAACCTCTTCCGCCTCCTCAAAAAGGACCTGGAATGTGTCGCCGTTGGA

guineapig1 TCTGTCACCTTCAACCTCCTCAGGCTGCTCACCCGGGACCTCAGATGTGTGGCCAGGCCA

hu_IFNL4_pseudo GCCACCACACCTGGCCTTTTCTGCCTCTCTATTTGGGACCTGAAGTGTGTGGCCAGTGGA

IFNL1 TCTGTCACCTTCAACCTCTTCCGCCTCCTCACGCGAGACCTCAAATATGTGGCCGATGGG

IFNL2 TCTGTCACCTTCAACCTCTTCCGCCTCCTCACGCGAGACCTGAATTGTGTTGCCAGTGGG

IFNL3 TCTGTCACCTTCAACCTCTTCCGCCTCCTCACGCGAGACCTGAATTGTGTTGCCAGCGGG

Ifnl1_mouse_pseudo TCTGTCACCCTCAACCTCTTTCGCCTGCTCACCTGGGACCTCAAGTATGTGGCCAGTGAA

Ifnl2_mouse TCTGTCACCTCCAACCTGTTTCGCCTGCTCACCCGGGACCTCAAGTGTGTGGCCAATGGA

Ifnl3_mouse TCTGTCACCTCCAACCTGTTTCAACTGCTCCTCCGGGACCTCAAGTGTGTGGCCAGTGGA

dog1 GACCTGTGCGTCTGA-------

dog2 GACCTGTGTGTG----------

dog3 GACCTGTGTGTC----------

guineapig1 GGGCACTGTGCTTGA-------

hu_IFNL4_pseudo GACCCGTGTGTCTGCCCCTGAG

IFNL1 AACCTGTGTCTGAGA-------

IFNL2 GACCTGTGTGTCTGA-------

IFNL3 GACCTGTGTGTCTGA-------

Ifnl1_mouse_pseudo GACCAGTGTGTC----------

Ifnl2_mouse GACCAGTGTGTCTGA-------

Ifnl3_mouse GACCAGTGTGTCTGA-------
